# Repressor element 1–silencing transcription factor drives the development of chronic pain states

**DOI:** 10.1097/j.pain.0000000000001633

**Published:** 2019-06-14

**Authors:** Fan Zhang, Sylvain Gigout, Yu Liu, Yiying Wang, Han Hao, Noel J. Buckley, Hailin Zhang, Ian C. Wood, Nikita Gamper

**Affiliations:** a Department of Pharmacology, Hebei Medical University, Shijiazhuang, China; b The Key Laboratory of Neural and Vascular Biology, Ministry of Education, Shijiazhuang, China; c The Key Laboratory of New Drug Pharmacology and Toxicology, Shijiazhuang, Hebei Province, China; d Faculty of Biological Sciences, University of Leeds, Leeds, United Kingdom; e Department of Psychiatry, University of Oxford, Oxford, United Kingdom

**Keywords:** Chronic pain, Epigenetics, K^+^ channel, Repressor element 1–silencing transcription factor, REST, NRSF

## Abstract

Chronic pain is an unmet clinical problem with vast individual, societal, and economic impact. Pathologic activity of the peripheral somatosensory afferents is one of the major drivers of chronic pain. This overexcitable state of somatosensory neurons is, in part, produced by the dysregulation of genes controlling neuronal excitability. Despite intense research, a unifying theory behind neuropathic remodelling is lacking. Here, we show that transcriptional suppressor, repressor element 1–silencing transcription factor (REST; neuron-restrictive silencing factor, NRSF), is necessary and sufficient for the development of hyperalgesic state after chronic nerve injury or inflammation. Viral overexpression of REST in mouse dorsal root ganglion (DRG) induced prominent mechanical and thermal hyperalgesia in vivo. Sensory neuron-specific, inducible *Rest* knockout prevented the development of such hyperalgesic state in 3 different chronic pain models. Genetic deletion of *Rest* reverted injury-induced hyperalgesia. Moreover, viral overexpression of REST in the same neurons in which its gene has been genetically deleted restored neuropathic hyperalgesia. Finally, sensory neuron specific *Rest* knockout prevented injury-induced downregulation of REST target genes in DRG neurons. This work identified REST as a major regulator of peripheral somatosensory neuron remodelling leading to chronic pain. The findings might help to develop a novel therapeutic approache to combat chronic pain.

## Abstract 2

Supplemental Digital Content is Available in the Text.

Transcriptional suppressor, repressor element 1-silencing transcription factor (REST, neuron-restrictive silencing factor), is necessary and sufficient for the development of chronic hyperalgesia after nerve injury or inflammation.

## 1. Introduction

Chronic pain constitutes an enormous ongoing health problem, yet, despite centuries of research and investment, the new treatments are slow coming and opioids are still a “gold standard.” Pathological changes within both peripheral and central nociceptive pathways contribute to the development and maintenance of chronic pain.^10^ One of the common peripheral causes of chronic pain is a pathological activity of a subset of peripheral nerves (nociceptors), which are normally silent and only become active in response to potentially damaging noxious stimuli.

Nerve injury triggers prominent morphological and epigenetic changes within afferent nerves. Mechanisms contributing to such remodelling include local inflammation,^29^ sympathetic sprouting,^7,24^ and a large-scale changes in gene expression in nociceptors, driving them into an overexcitable state.^3,28,41^ Control of gene expression is orchestrated by transcription factors that chemically modify DNA or histones (reviewed in Ref. 28). One of such transcription factors upregulated in sensory neurons after nerve injury^30,38,39,43,44^ and contributing to chronic pain development is the repressor element 1–silencing transcription factor REST (also known as neuron-restrictive silencing factor).^38,39,43,44^ REST is a zinc-finger transcription factor mediating both short- and long-term transcriptional suppression of target genes containing RE1 binding sites within their promotor regions. REST forms a large complex with other enzymes and regulators, including CoREST, LSD1, BHC80, and BRAF35^26–28^; it also recruits additional enzymes, including MeCP2, histone deacetylases HDAC1/2, and the histone methyltransferase G9a. Acting together, these elements of the complex suppress gene expression by removing active histone marks, such as H3K4 methylation or various histone acetylations and adding repressive histone marks such as H3K9 methylation.^1,2,27,28^

The main physiological role of REST was initially considered to be in a suppression of neuronal genes in non-neuronal cells.^6,31^ While now REST is believed to play some important roles in several neural disorders and brain ageing, the expression of REST in healthy neurons is generally low.^35,40^ However, expression of REST in peripheral somatosensory neurons was shown to be elevated in neuropathic^30,38,39,43^ and bone cancer pain^44^ models. These studies also revealed that the expression of some of REST target genes, including *Kcnd3*, *Kcnq2*, *Scn10a*, and *Oprm1* (coding for Kv4.3, Kv7.2, Na_v_1.8 ion channels, and μ-opioid receptors, respectively) was concurrently repressed. These changes were further correlated with the development of hyperalgesia, C-fiber overexcitability and a reduction in the efficacy of opioid analgesia.^30,38,39,44^ Intrathecal injection of *Rest* antisense oligonucleotides or siRNA alleviated some of these transcriptional and behavioural changes^38,43,44^ suggesting that REST is an important driver of these pathological responses.

Although a proalgesic role of REST in peripheral somatosensory system is being increasingly recognised, the overall contribution of REST to the development of chronic pain remains unclear. In this study, we used in vivo viral gene delivery and sensory neuron restricted, inducible *Rest* knockout to demonstrate that REST is necessary and sufficient for the development of chronic pain.

## 2. Methods

### 2.1. Animals

Animal experiments performed in Hebei Medical University were in accordance with the Animal Care and Ethical Committee of Hebei Medical University, (Shijiazhuang, China) under the International Association for the Study of Pain (IASP) guidelines. Animal experiments performed in the University of Leeds were in accord with the regulations of the Research Ethics Committee of the Faculty of Biological Sciences at the University of Leeds and under the provisions of the UK Animals (Scientific Procedures) Act 1986. For the generation of *Rest*^loxP/loxP^/AvCreER-T2 mouse line, *Rest*^loxP/loxP^ mice^34^ were crossed with the AvCreER-T2 mice,^20^ kindly provided by Prof John Wood (UCL). To induce Cre recombinase activity, 5 daily intraperitoneal injections of tamoxifen (2 mg in 0.2 mL of sunflower oil) were given to the mice. In later experiments, the amount of tamoxifen injections was reduced to 4 as genotyping confirmed that it was sufficient to induce the knockout. In the experiments with knockout mice, both male and female animals were used.

### 2.2. Viral gene delivery to dorsal root ganglion

AAV2/9-CMV-REST, AAV2/9-CMV-NULL, AAV2/9-DIO-REST, and AAV2/9-DIO-NULL virions were produced by Hanbio Biotechnology Co, Ltd AAV2/9 virion injections into the right-side L4 dorsal root ganglion (DRG) of C57BL/6 male mice or transgenic mice of either sex were performed as previously described.^14^ Briefly, in deeply anesthetised mice (sodium pentobarbital, 80-100 mg/kg; intraperitoneally), L4 DRGs were exposed by removal of both spinous and transverse processes of the vertebra bone. The microinjector (Hamilton Company, Reno, NV) was inserted into the ganglion to a depth of 500 μm from the exposed surface. The virion solution (1.1-1.2 × 10^12^ vg/mL; 5 μL) was injected slowly, and the needle was removed 2 minutes after the injection was complete. The muscles overlying the spinal cord were loosely sutured together, and the wound was closed. Thermal and mechanical sensitivity tests were performed before the injection and at a weekly interval after the injection. At 28 days after injection, animals were sacrificed, L4 DRGs extracted and tested for expression of Myc-tagged REST using immunohistochemistry. Animals developing signs of distress were sacrificed.

### 2.3. Chronic pain models

To induce chronic inflammatory pain, a complete Freund's adjuvant (CFA, 20 μL) was injected into the plantar surface of the right hind paw of the mice as described.^14^ Partial sciatic nerve ligation (PSNL) was performed as described.^30^ Briefly, mice were anesthetised with 2% vol/vol isoflurane; the left sciatic nerve was exposed at midthigh level and cleared of surrounding connective tissues. A 6-0 Prolene suture (Ethicon Ltd, Edinburgh, United Kingdom) was inserted into the nerve with a 3/8 curved, reversed-cutting needle, and tightly ligated, so that the dorsal third/half of the nerve was held within the ligature. A small cut was made approximately 5 mm below the ligature. The wound was then closed with sutures. In sham-operated mice, the left sciatic nerve was exposed but untouched.

The spared nerve injury (SNI) was performed following the procedure described previously.^8^ Briefly, under deep anaesthesia (sodium pentobarbital, 80-100 mg/kg, intraperitoneally), 3 distal branches of the right sciatic nerve were exposed carefully without damaging the muscle bundles. The common peroneal and the tibial nerves were exposed and tightly ligated with 5–0 silk and transected distal to the ligation, removing a 2- to 4-mm length of each nerve. Great care was taken to avoid any contact with or stretching of the intact sural nerve. The wound was closed with sutures.

### 2.4. Behavioral tests

In all tests, animals were habituated to the testing environment for at least 3 hours before testing. Mechanical withdrawal threshold was measured by a set of von Frey filaments (Stoelting Co, Chicago, IL) with a calibrated range of bending force. Each animal was placed into a plastic cage with a wire mesh bottom. A single filament was applied perpendicularly to the plantar surface of hind paw. A response was considered as positive when the animal withdrew sharply its paw; the same stimulus was applied for 5 times with an interval of 5 seconds. In the withdrawal threshold measurements, the threshold was recorded when 3 clear withdrawal responses of 5 applications were observed with a given filament. For the 50% withdrawal threshold method, the 50% threshold was determined using the up and down method according to the protocol described in Ref. 5. The 50% threshold value was determined according to the following equation: 50% withdrawal threshold = (10^[*X*f + *k*δ]^)/10,000; where *X*_f_ is a value (in log units) of the final von Frey filament used; *k* is a tabular value for the pattern of positive/negative responses; and δ is a mean difference (in log units) between stimuli.^5^

Thermal withdrawal latency was tested by a radiant heat lamp source (PL-200, Taimeng Co, Chengdu, China). The intensity of the radiant heat source was maintained at ∼25%. Animals were placed individually into Plexiglas cubicles with a transparent glass surface. The light beam from radiant heat lamp, located below the glass, was directed at the plantar surface of hind paw. The time was recorded from the onset of radiant heat stimulation to withdrawal of the hind paw. Three trials with an interval of 5 minutes were made for each paw/animal, and scores from 3 trials were averaged.

Cold sensitivity was assessed in the cold plate test using the cold plate apparatus (hot/cold plate, Bioseb, France). Mice were placed on a 1°C cold plate, and the latency to the first brisk hind paw lift or flicking/licking of the hind paw or jumping was measured, with a cutoff time of 120 seconds. Number of flinches/jumps within 120 seconds was also measured.

Whenever practical, behavioural experiments were performed by an experimenter blinded to genetic background of animals or treatment schedule.

### 2.5. Immunohistochemistry

L4 DRGs were dissected, postfixed in 4% paraformaldehyde at 4°C for 2 hours, and cryoprotected in 20% sucrose in PBS at 4°C overnight. Before staining, 10-μm sections were postfixed with 4% paraformaldehyde in PBS for 10 minutes. After blocking in 10% goat serum and 0.3% TritonX-100 in PBS for 1 hour at room temperature, the sections were incubated at 4°C overnight with mouse primary c-Myc antibody (Invitrogen, Carlsbad, CA; 1:50) or rabbit primary antibody against REST (Abcam, Cambridge, United Kingdom; 1:50). On the second day, the sections were washed in PBS 3 times and then incubated with rabbit anti-mouse secondary antibody (1:100, Jackson ImmunoResearch, Ely, United Kingdom) at room temperature for 2 hours. The sections were then washed with PBS 3 times and placed on microscope slides in Vectashield with DAPI (Vector Laboratories, Peterborough, United Kingdom). Staining was visualized using a confocal fluorescent microscope (LSM700, Zeiss, Oberkochen, Germany).

### 2.6. Single-cell RT-PCR

Dorsal root ganglia were extracted and dissociated using a collagenase-dispase method as described.^13^ After the dissociation, neurons were aspirated (under a microscope) into a patch pipette using a conventional patch-clamp setup with negatively pressurised pipette holder. The electrode tip was then quickly broken into a 0.2-mL PCR tube containing 0.7 µL of oligo-dT (50 mM), 1 µL of dNTP mixture (10 mM), 0.5 µL of MgCl_2_ (25 mM), 0.7 µL of RNaseOUT (40 U/µL), and 1.4 µL of DEPC-treated water; the mixture was heated to 65°C for 5 minutes and then placed on ice for 1 minute. Single-strand cDNA was synthesized from the cellular mRNA by adding 0.5 µL of RT buffer, 1.5 µL of MgCl_2_ (25 mM), 1 µL of DTT (1 M), 0.5 µL of RNaseOUT (40 U/µL), and 1 µL of SuperScript III RT (200 U/µL) and then incubating the mixture at 55°C for 50 minutes followed by 85°C for 5 minutes. Synthesis of single-cell cDNA was performed using a C1000 Touch thermal cycler-CFX96 Real-time PCR (BIO-RAD, Hercules, CA). First strand synthesis was executed at 95°C (1 minute) followed by 40 cycles (95°C for 50 seconds, 60°C for 50 seconds, and 72°C for 55 seconds) and a final 10 minutes elongation at 72°C by adding the specific “outer” primer pairs (suppl. Table 1, available at http://links.lww.com/PAIN/A830) into each PCR tube (final volume 60 μL). Then, 2.5 μL of the product of the first PCR was used in the second amplification round by using specific “inner” primers (final volume 25 μL; suppl. Table 1, available at http://links.lww.com/PAIN/A830). The second amplification round consisted of heating the samples to 95°C (1 minute) followed by 40 cycles (95°C for 50 seconds, 60°C for 50 seconds, and 72°C for 55 seconds) and 10-minute elongation at 72°C. The products of the second PCR were analysed in 2% agarose gels and stained with ethidium bromide. SuperScript III First-Strand Synthesis System Kit and GoTaq Green Master Mix were obtained from Takara-Clontech (Invitrogen, Carlsbad, CA) and Promega (Madison, WI), respectively.

### 2.7. Statistics

All data are given as mean ± SEM. Differences between groups were assessed by two-way repeated-measures ANOVA with Tukey post-test. Differences were considered significant at *P* ≤ 0.05. Single-cell RT-PCR data (detection incidence) were analysed using Fisher's exact test. Statistical analyses were performed using OriginPro 9.1 (Originlab Corporation, Northampton, MA). *, **, *** indicate significant difference from the appropriate control with *P* ≤ 0.05, *P* ≤ 0.01, or *P* ≤ 0.001.

## 3. Results

### 3.1. Overexpression of REST in peripheral afferent fibers induces sustained hyperalgesia

**Figure 1.**
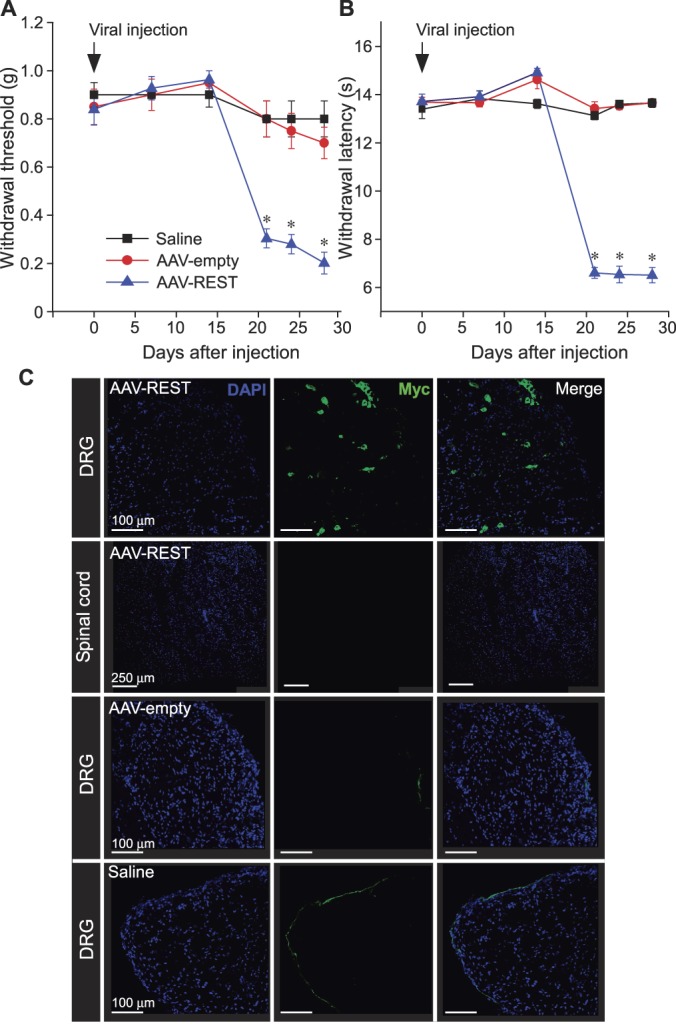
Overexpression of REST in DRG induces hyperalgesic state in mice. (A) and (B) Injection of AAV2/9 coding for Myc-tagged REST into the DRG of the mice in vivo (1.2 × 10^12^ vg/mL; 5 μL) produced strong mechanical (A) and thermal (B) hyperalgesia (blue symbols, lines; n = 10) as compared to saline-injected (black symbols, lines; n = 6) or empty virus-injected (red symbols, lines; n = 8) animals. *P < 0.05 (two-way repeated-measures ANOVA with Tukey post-hoc test). (C) Immunohistochemical analysis of Myc expression in L4 DRG (top row) or the corresponding segment of the spinal cord (second row from the top) of the mice received AAV2/9-REST. Also shown DRGs of mice received empty AAV2/9 particles (second raw from the bottom) or saline (bottom row). Blue staining represents DAPI nuclear labelling, green staining represents Myc. Three mice (5-6 sections) were analysed in each row. ANOVA, analysis of variance; DRG, dorsal root ganglion.

We first tested whether overexpression of REST in mouse DRG neurons is sufficient to generate hyperalgesia in mice. To this end, we designed adeno-associated virions coding for Myc-tagged REST (AAV2/9-REST). In accord with our previous data showing that overexpression of REST in DRG neurons makes them overexcitable,^25^ unilateral injection of AAV2/9-REST into the DRG of control mice (see Methods) resulted in the development of prominent mechanical (Fig. 1A) and thermal (Fig. 1B) hyperalgesia in vivo. The mechanical hyperalgesia was the most pronounced with withdrawal threshold (von Frey test, see Methods) changing from 0.84 ± 0.07 g before the injection to 0.20 ± 0.05 g at 28 days after the injection (n = 10; *P* ≤ 0.05). Thermal withdrawal latency (Hargreaves test, see Methods) changed from 13.7 ± 0.3 seconds before the injection to 6.5 ± 0.3 seconds at 28 days after (n = 10; *P* ≤ 0.05). Injection of empty AAV2/9 virions produced no effect in either of the tests (Figs. 1A and B). There was about 2-week delay in the onset of hyperalgesia, likely to be due to the time required for the in vivo viral transduction to take place.^45^ Mice injected with AAV2/9-REST displayed visible change of gait (suppl. Movie 1, available at http://links.lww.com/PAIN/A826), likely to be a consequence of the protective behaviour towards the hyperalgesic paw. Control mice injected with empty virions did not demonstrate any noticeable change in gait (suppl. Movie 2, available at http://links.lww.com/PAIN/A827). At 28 days after the viral injection, animals were sacrificed and L4 DRGs were analysed for the presence of exogenous REST using anti-Myc antibody. No specific antibody binding was detected in DRGs of mice injected with saline or an empty AAV2/9, whereas multiple DRG cell bodies from the AAV2/9-REST–injected animals displayed fluorescent staining indicative of the exogenous expression of the Myc-tagged REST (Fig. 1C). Quantification of these data revealed that approximately 25% of DRG neurons in the ipsilateral L4 DRG of the AAV2/9-REST–injected mice were Myc-positive (suppl. Fig. 1, available at http://links.lww.com/PAIN/A830). It must be noted that nuclear DAPI staining seen in Figure 1C identifies all cells in the ganglia (including abundant satellite glia and other cell types), not only neurons; neurons were identified by their shape during confocal imaging. No fluorescence was detected in the corresponding sections of the spinal cord (Fig. 1C, second row from the top), confirming the accuracy of the viral gene delivery. These experiments provide clear evidence that increase of REST expression in DRG neurons is in itself sufficient to produce chronic pain–like phenotype in mice.

### 3.2. Sensory neuron-specific, inducible knockout of Rest prevents development of chronic pain after peripheral nerve injury or inflammation

**Figure 2.**
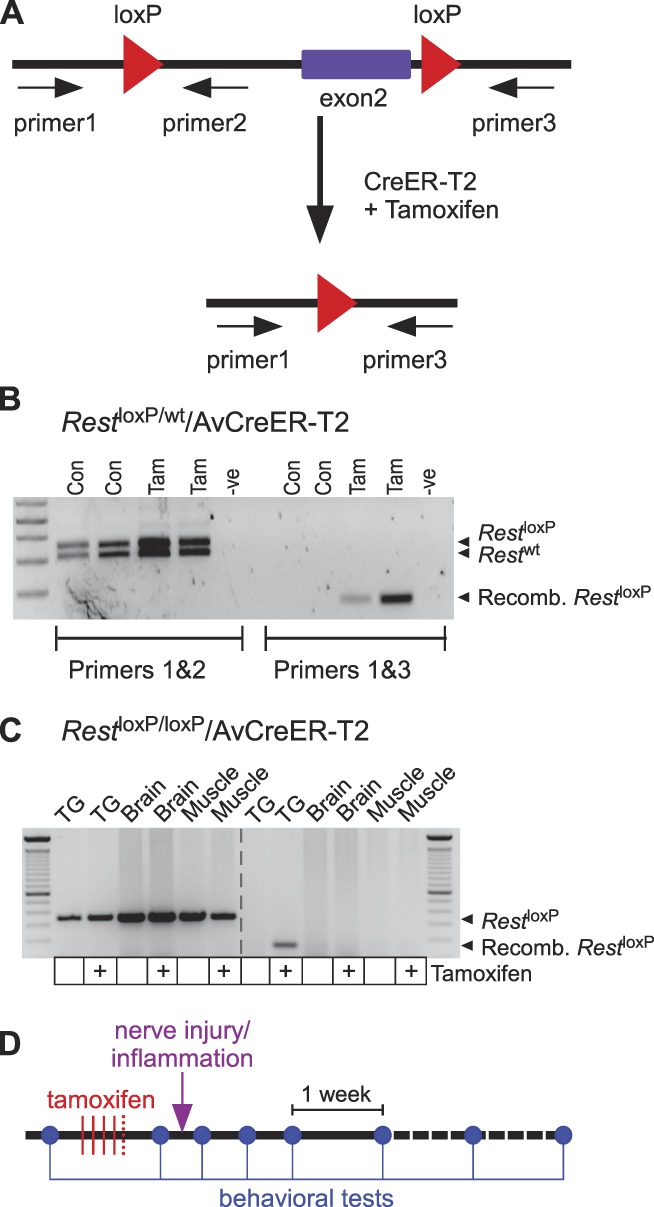
Tamoxifen induced *Rest* Knock-out. (A) Representation of the modified *Rest*fl allele showing the loxP sites flanking exon 2. CreER expression is driven by the advillin promoter and is restricted to peripheral sensory neurons. In the presence of tamoxifen, CreER activity is induced resulting in the loss of *Rest* exon 2, abolishing REST expression. (B) PCR genotyping of DNA extracted from DRG of vehicle (Con) or Tamoxifen (Tam)-treated *Rest*^loxP/wt^/AvCreER-T2 mice. (C) PCR genotyping of DNA extracted from trigeminal sensory ganglia (TG), brain, and muscle of tamoxifen- (+) or vehicle-treated *Rest*^loxP/loxP^/AvCreER-T2 mice. Panels (B) and (C) are representative of 3 independent experiments. (D) Schematic of experiments in which tamoxifen-induced *Rest* deletion was performed before establishment of chronic pain model; timing of behavioural tests (von Frey, Hargreaves, or cold plate tests, see Methods) is indicated in blue. DRG, dorsal root ganglion.

We next decided to test how deletion of *Rest* specific to sensory neurons might affect the development of chronic pain. We crossed the floxed *Rest* mice^34^ with the mice expressing tamoxifen-inducible CreER-T2 recombinase under control of advillin promoter^20^ (advillin is expressed almost exclusively in peripheral sensory neurones^17^). The resultant *Rest*^loxP/loxP^/AvCreER-T2 line (Fig. 2A) allows for *Rest* to be knocked down in adult animals and in sensory neurons specifically. Excision of *Rest* in DRG neurons by tamoxifen (2 mg; 4 or 5 daily intraperitoneal injections) was confirmed by PCR (Fig. 2B). We also confirmed that nonspecific knockout of *Rest* was not detectable in the brain or muscle tissue samples (Fig. 2C); trigeminal sensory ganglia were used for genotyping in this case to spare DRGs for other experiments. We next set out to test how conditional deletion of *Rest* would affect development of chronic pain in neuropathic or chronic inflammation pain models; the schedule of a typical experiment is given in Figure 2D.

**Figure 3.**
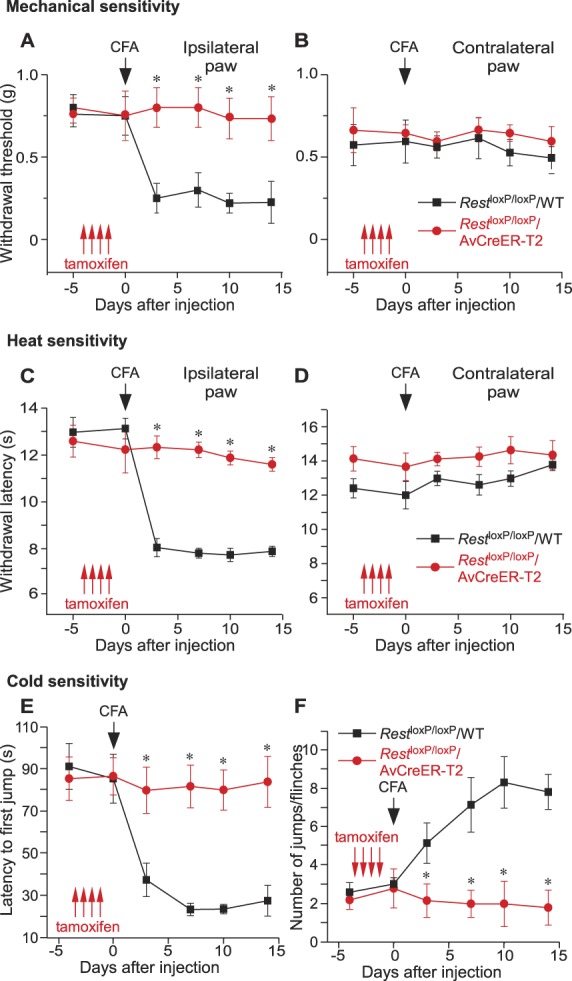
Deletion of *Rest* prevents development of chronic inflammatory hyperalgesia. Injection of tamoxifen (at times indicated by red arrows) prevented the development of mechanical (A) and thermal (C) hyperalgesia in *Rest*^loxP/loxP^/AvCreER-T2 (red symbols, lines; n = 6) but not in *Rest*^loxP/loxP^/WT (black symbols, lines; n = 6) mice, which received a hind paw injection of the Complete Freund's Adjuvant (CFA, 20 μL) at the time indicated by black arrow. Measurements were performed on the ipsilateral paw. (B) and (D) Show experiments similar to these shown in (A) and (C) but performed on the contralateral paw. *P < 0.05 (n = 6 for each group in (B) and (D); two-way repeated-measures ANOVA with Tukey post hoc test). (E) and (F) Cold sensitivity measurements (cold plate test, 1°C; see Methods), following the same experimental paradigm as in panels (A–D). Latency to first jump/paw flinch and number of jumps/flinches within a 120-second trial are shown in (E) and (F), respectively. *Rest*^loxP/loxP^/AvCreER-T2: n = 5; *Rest*^loxP/loxP^/WT: n = 6. *P < 0.05 (two-way repeated-measures ANOVA with Tukey post hoc test). ANOVA, analysis of variance.

First, we knocked out *Rest* before inducing chronic inflammation using a hind paw injection of a CFA.^19^ Consistent with the previous reports,^14,19^ CFA induced prominent hyperalgesia to mechanical, hot and cold stimuli (Figs. 3A–F) on the ipsilateral, but not on the contralateral to CFA injection side in the tamoxifen-injected *Rest*^loxP/loxP^/WT mice lacking the Cre (control). The hyperalgesia persisted for at least 2 weeks of observation. Strikingly, tamoxifen-injected *Rest*^loxP/loxP^/AvCreER-T2 mice displayed complete lack of hyperalgesia to either type of stimulation (Figs. 3A–F). These results suggest that REST is instrumental in the establishment of chronic inflammatory pain state. Of note is the fact that CFA-induced paw edema was visually not different between the tamoxifen-injected *Rest*^loxP/loxP^/WT and *Rest*^loxP/loxP^/AvCreER-T2 mice (suppl. Fig. 2, available at http://links.lww.com/PAIN/A830), suggesting that in the absence of *Rest*, the inflammatory response to CFA still occurs.

**Figure 4.**
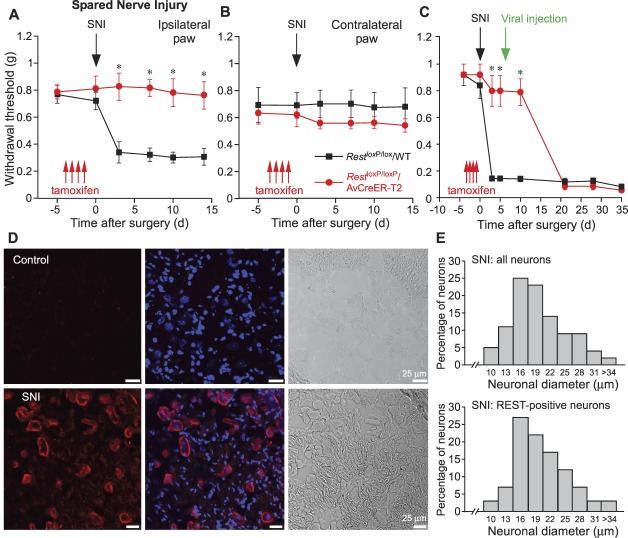
Deletion of *Rest* prevents development of hyperalgesia in spared nerve injury model of neuropathic pain. (A) Mechanical sensitivity (withdrawal thresholds) in mice measured at specific days relative to spared nerve injury (SNI, black arrow; schematically depicted in suppl. Fig. 3, available at http://links.lww.com/PAIN/A830); *Rest*^loxP/loxP^/AvCreER-T2 (red symbols, lines; n = 6), *Rest*^loxP/loxP^/WT (Control; black symbols, lines; n = 6). Mice were injected with tamoxifen daily over a 4-day period before injury (red arrows). Measurements were performed on the ipsilateral paw. (B) Similar to (A) but measurements were performed on the contralateral paw; n = 6 for both groups. (C) *Rest*^loxP/loxP^/AvCreER-T2 (red symbols, lines; n = 5) and *Rest*^loxP/loxP^/WT (Control; black symbols, lines; n = 5) were injected with tamoxifen (red arrows) with subsequent SNI surgery (black arrow). At day 6 after SNI, *Rest*^loxP/loxP^/AvCreER-T2 mice were DRG-injected with AAV2/9-DIO-REST virions (1.2 × 10^12^ vg/mL; 5 μL), while *Rest*^loxP/loxP^/WT mice were injected with AAV2/9-DIO-empty virions (1.1 × 10^12^ vg/mL; 5 μL). Mechanical withdrawal threshold was measured on the ipsilateral paw at times indicated. *P < 0.05 (two-way repeated-measures ANOVA with Tukey post hoc test). (D) Immunohistochemical detection of REST in DRG of *Rest*^loxP/loxP^/WT mice (without tamoxifen injection) in control conditions (top row) or 2 weeks after the SNI (bottom row). (E) Top panel: cell size distribution of all neurons within the DRG of *Rest*^loxP/loxP^/WT mice (without tamoxifen injection) 2 weeks after the SNI (3 mice, 6 sections; n = 133). Bottom panel: cell size distribution of REST-positive neurons in the same sections (3 mice, 6 sections; n = 61). The neuronal diameter was measured to the nearest micrometer and is presented as a frequency distribution. In the top panel, all neuronal cell bodies with distinguishable nuclei were quantified; in the bottom panel, only REST-positive neurons were quantified. DRG, dorsal root ganglion.

Next, we tested the effect of *Rest* deletion in 2 different models of neuropathic pain (suppl. Fig. 3, available at http://links.lww.com/PAIN/A830): SNI^8^ and PSNL.^32^ As before, we compared the tamoxifen-injected *Rest*^loxP/loxP^/WT mice lacking the Cre (control) with the tamoxifen-injected *Rest*^loxP/loxP^/AvCreER-T2 mice. As in the case with the CFA injection, SNI induced prominent mechanical hyperalgesia, which persisted for the entire period of observation (2 weeks after surgery in this case) in the ipsilateral but not the contralateral side of the control mice. However, the hyperalgesia was completely absent in the *Rest*^loxP/loxP^/AvCreER-T2 mice (Figs. 4A and B). Control mice with SNI displayed protective behaviour towards the injured paw and changed gait (suppl. Movie 3, available at http://links.lww.com/PAIN/A828), but this behaviour was not evident in the *Rest*^loxP/loxP^/AvCreER-T2 mice with the SNI injury (suppl. Movie 4, available at http://links.lww.com/PAIN/A829).

In the next experiment, we tested whether we can offset *Rest* deletion in *Rest*^loxP/loxP^/AvCreER-T2 mice with viral overexpression and whether such a manoeuvre would reinstate the development of neuropathic hyperalgesia. For this, we designed AAV2/9-DIO-REST virions using the double-floxed inverse open reading frame (DIO) approach. This design restricted the expression of REST to cells expressing Cre recombinase.^33^ The experiment had the following rationale: after the tamoxifen injection, the DRG neurons in *Rest*^loxP/loxP^/AvCreER-T2 mice will start expressing Cre; this will cause REST deletion but on the other hands, this would also make these neurons specifically susceptible to infection by the AAV2/9-DIO-REST virions. After the injection of tamoxifen, *Rest*^loxP/loxP^/WT (control) and *Rest*^loxP/loxP^/AvCreER-T2 mice were subjected to SNI injury, and 6 days later, animals were DRG-injected with the AAV2/9-DIO-empty or AAV2/9-DIO-REST virions, respectively (Fig. 4C). *Rest*^loxP/loxP^/WT mice developed obvious mechanical hyperalgesia immediately after SNI. Consistent with earlier experiments (Fig. 4A), *Rest*^loxP/loxP^/AvCreER-T2 mice did not develop noticeable hyperalgesia after the SNI; however, AAV2/9-DIO-REST injection resulted in a complete recovery of the hyperalgesic phenotype (Fig. 4C). Again, there was a delay in the onset of hyperalgesia, but 2 weeks after the viral REST delivery, the *Rest*^loxP/loxP^/AvCreER-T2 mice with SNI became as hyperalgesic as the control (AAV2/9-DIO-empty virus-injected *Rest*^loxP/loxP^/WT) animals. This experiment further supported the hypothesis that rising levels of REST in sensory neurons specifically is necessary for the development of neuropathic pain.

We also tested the expression of REST in DRG neurons after SNI. Consistent with previous reports,^30,38,42^ REST immunofluorescence was barely detectable in the DRG of control animals (*Rest*^loxP/loxP^/WT; Fig. 4D, upper panel). However, 2 weeks after SNI, many DRG neurons were strongly labelled with anti-REST antibody (Fig. 4D, lower panel). Size distribution of REST-positive neurons in SNI animals was broadly consistent with the general size distribution of neurons in the same DRG (Fig. 4E), and this, in turn, was consistent with previous literature.^22^ Hence, it is likely that REST upregulation induced by injury affects all types of neurons in DRG.

**Figure 5.**
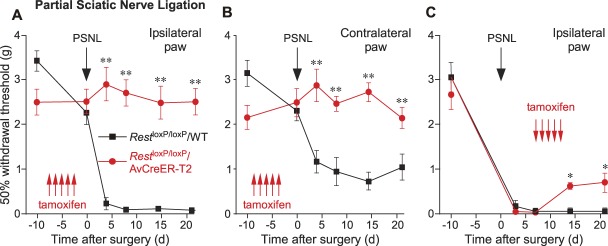
Deletion of *Rest* prevents development of hyperalgesia in partial sciatic nerve ligation model of neuropathic pain. (A) and (B) Mechanical sensitivity (withdrawal thresholds) in mice measured at specific days relative to partial sciatic nerve ligation (PSNL, black arrow; schematically depicted in suppl. Fig. 3, available at http://links.lww.com/PAIN/A830) in *Rest*^loxP/loxP^/AvCreER-T2 (red symbols, lines; n = 10) and *Rest*^loxP/loxP^/WT (Control; black symbols, lines; n = 8). Mice were injected with tamoxifen daily over a 5-day period before injury (red arrows). (C) An experiment similar to that shown in the panel A, but tamoxifen was injected (at times indicated by red arrows) after the PSNL surgery (time of the surgery is indicated by the black arrow; n = 10 for both groups). *P < 0.05 (two-way repeated-measures ANOVA with Tukey post hoc test). ANOVA, analysis of variance.

Qualitatively similar results were obtained with the PSNL injury model. Consistent with previous reports, PSNL injury resulted in a prominent mechanical hyperalgesia on the injured (ipsilateral) side, as well as a noticeable “mirror” mechanical hyperalgesia in the contralateral paw^30,32^ in the control mice (tamoxifen-injected *Rest*^loxP/loxP^/WT; Figs. 5A and B). Hyperalgesia was absent in the tamoxifen-injected *Rest*^loxP/loxP^/AvCreER-T2 mice (on either the ipsilateral or contralateral paws; Figs. 5A and B). This striking difference was maintained for the entire observation period (21 days after injury in this case; Figs. 5A and B). In the next experiment, we inverted the order of *Rest* deletion and the neuropathic injury: we performed PSNL, allowed the development of measurable hyperalgesia for 1 week (Fig. 5C), and then applied tamoxifen (daily injections on days 7th-11th after the PSNL surgery; see Methods). Mechanical sensitivity was then tested again at days 14 and 21 after the surgery. Tamoxifen injection did not produce any effect in control mice (i.e., the PSNL-induced hyperalgesia persisted without a change in these animals). Knockout of *Rest*, in *Rest*^loxP/loxP^/AvCreER-T2 mice with already developed PSNL-induced mechanical hyperalgesia resulted in a significant alleviation of hyperalgesia by 2 to 9 days after the final injection of tamoxifen, although withdrawal threshold did not return to presurgery levels completely during this period (Fig. 5C). These data suggest that PSNL-induced hyperalgesia can be at least partially reversed by removal of REST. In some experiments shown in Figures 5A–C, *Rest*^loxP/loxP^/AvCreER-T2 mice displayed somewhat lowered baseline withdrawal thresholds; the difference did not reach significance with the statistical test used.

Of note, in the absence of tamoxifen, *Rest*^loxP/loxP^/AdvCreER-T2 mice had normal mechanical threshold sensitivity and developed hyperalgesia after nerve injury (PSNL) similarly to the WT littermates (suppl. Fig. 4, available at http://links.lww.com/PAIN/A830). Taken together, the results presented in Figures 3–5 clearly indicate that (1) sensory neuron-specific *Rest* knockout prevents development of hyperalgesia in 3 different chronic pain models; (2) neuropathic hyperalgesia can be rescued in nerve-injured animals by overexpressing REST in otherwise *Rest*-deficient sensory afferents; (3) under conditions where the nerve injury–induced hyperalgesia is allowed to develop, deletion of *Rest* produces an antialgesic effect.

Upregulation of REST after peripheral nerve injury has been shown to correlate with the downregulation of some of its target genes in DRG, including *Kcnd3*,^39^ *Kcnq2*,^30^ *Scn10a*,^38^ and *Oprm1*^38,44^; these genes are coding for Kv4.3, Kv7.2, Na_v_1.8 ion channels and μ-opioid receptors, respectively. Thus, we used single-cell RT-PCR (see Methods) to test the following: (1) if we can detect upregulation of REST and reduction in the expression of its targets after nerve injury (SNI in this case) on a single cell level; (2) if genetic deletion of *Rest* could prevent injury-associated changes in the expression of REST targets.

**Figure 6.**
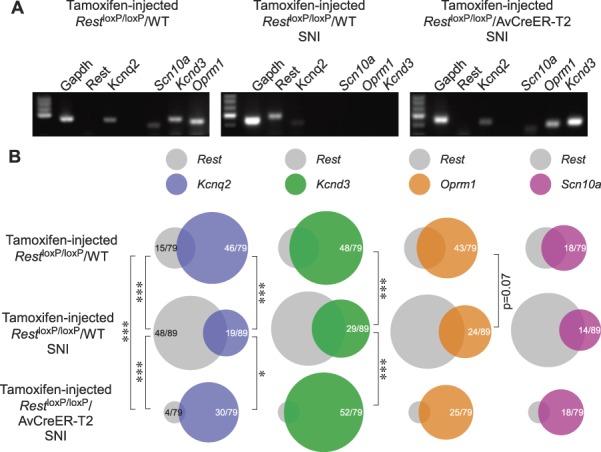
Deletion of *Rest* prevents nerve injury induced downregulation of REST target genes. (A) Examples of single-cell RT-PCR detection of *Gapdh*, *Rest*, *Kcnq2*, *Scn10a*, *Oprm1*, and *Kcnd3* in individual DRG neurons dissociated from either the naïve, tamoxifen-injected *Rest*^loxP/loxP^/WT mice (left), or tamoxifen-injected *Rest*^loxP/loxP^/WT mice 2 weeks after SNI injury (middle), or tamoxifen-injected *Rest*^loxP/loxP^/AvCreER-T2 mice 2 weeks after SNI injury (right). (B) Venn diagrams depicting incidence of detection of *Rest*, *Kcnq2*, *Scn10a*, *Oprm1*, and *Kcnd3*, in DRG neurons dissociated from the 3 category of mice, as indicated. Also indicated is the degree of overlap between the detection of *Rest* and either of the other gene in a single cell. Asterisks indicate difference between groups linked by the line connectors, as tested with Fisher's exact test. Numbers within each pie chart indicate number of positive cells out of total number of cells analysed; data for *Rest* are the same in each column. More detailed quantification is presented in suppl. Table 2 (available at http://links.lww.com/PAIN/A830). DRG, dorsal root ganglion.

Two weeks after the induction of SNI, DRGs were dissociated and single DRG neurons collected for analysis immediately (see Methods). For each gene, we quantified the incidence of detection (percentage of positive DRG neurons from the total cells tested); these data are presented in Figure 6 and suppl. Table 2 (available at http://links.lww.com/PAIN/A830). In control mice (tamoxifen-injected *Rest*^loxP/loxP^/WT) after SNI, there was a significant increase in *Rest*-positive neurons (from 19% to 53%; *P* ≤ 0.001), accompanied by the significant decrease in the incidence of *Kcnq2* (from 58% to 21%; *P* ≤ 0.001) and *Kcnd3* (from 61% to 33%; *P* ≤ 0.001). Incidence of neurons expressing *Oprm1* and *Scn10a* was also lower (from 41% to 27%; *P* = 0.07 and from 23% to 16%; *P* = 0.3), but this difference did not reach statistical significance (Fig. 6; suppl. Table 2, available at http://links.lww.com/PAIN/A830). The tamoxifen-injected *Rest*^loxP/loxP^/AvCreER-T2 mice differed from the control in 2 aspects: (1) *Rest* was undetectable in most of the cells even after the SNI injury; (2) there was no significant reduction in the incidence of their REST target genes we tested (Fig. 6; suppl. Table 2, available at http://links.lww.com/PAIN/A830). While in some *Rest*-positive neurons other genes of interest were also found (Fig. 6B), it is of note that in most “de novo” *Rest*-positive neurons in the tamoxifen-injected *Rest*^loxP/loxP^/WT mice after SNI, the REST target genes tested were not found. Taken together, these data support our hypothesis that knocking down *Rest* prevents nerve injury–induced remodelling of sensory neurons.

It has to be noted that AAV2/9 injections, CFA, and SNI experiments were performed in the Hebei Medical University while PSNL experiments were performed at the University of Leeds. Mechanical sensitivity was assessed using slightly different methods (mechanical withdrawal threshold measurement was used at the Hebei Medical University, and the 50% threshold method was used at the University of Leeds), which gave somewhat different absolute values (but consistent with the reported range^9^). Nevertheless, the outcome of these tests obtained in 2 laboratories was virtually identical: genetic deletion of *Rest* abolished the development of chronic pain in neuropathic and chronic inflammation pain models.

## 4. Discussion

In this study, we demonstrated that transcriptional suppressor REST is necessary and sufficient for the development of hyperalgesic state after chronic nerve injury or inflammation. Unilateral overexpression of REST in DRG of wild-type mice induced prominent mechanical and thermal hyperalgesia in the absence of actual injury or inflammation. By contrast, sensory neuron-specific, inducible *Rest* knockout effectively prevented the development of such hyperalgesic state in 3 different models of chronic pain (CFA, SNI, and PSNL). Importantly, hyperalgesia was reinstated in nerve-injured mice with Cre-mediated *Rest* KO by Cre-dependent viral overexpression of REST in DRG (Fig. 4C). In addition, genetic deletion of *Rest* in DRG (Fig. 5C) partially reversed injury-induced hyperalgesia. Sensory neuron-specific *Rest* knockout also prevented injury-induced downregulation of REST target genes in DRG neurons, as tested with single-cell RT-PCR. In this study, we did not directly measure binding of REST to the *Kcnd3*, *Kcnq2*, *Scn10a*, or *Oprm1* promoter regions, but such binding has been demonstrated in early work by us^25^ and others.^38,39^ Taken together, our data identified REST as one of the key transcriptional regulators of peripheral somatosensory neuron remodelling leading to chronic pain.

Of note is the fact that while deletion of *Rest* before the onset of either of the 3 chronic pain models used in this study virtually abolished the development of hyperalgesia, *Rest* deletion 1 week after the PNSL surgery produced only partial alleviation of mechanical hyperalgesia (Fig. 5C). The reasons for this difference will have to be established, but one explanation could be in that the initial upregulation of REST, induced during the first week after the nerve injury, could trigger a cascade of long-term downstream changes that cannot be promptly and entirely reversed at this time point by the removal of *Rest*. On the other hand, if *Rest* is already absent at the onset of the nerve injury, then these downstream changes are prevented altogether. Future studies should test if this hypothesis is correct.

It has to be pointed out that (1) not all REST targets downregulated in chronic pain models are antiexcitatory and (2) not all REST targets are found downregulated in chronic pain models. In regards to the first point above, *Scn10a*, coding for the excitatory voltage-gated Na^+^ channel Na_v_1.8 has an RE1 consensus site and was found downregulated in PSNL model.^38^ However, downregulation of Na_v_1.8 is perhaps not a universal feature of chronic pain conditions and could be compensated by the upregulation of Na_v_1.8 activity^4^ and enhanced expression of other voltage-gated Na^+^ channels (eg, Na_v_1.3) in DRG.^42^ Regarding the second point, indeed chronic pain associated upregulation of genes encoding L- and T-type Ca^2+^ channels, vasoactive intestinal peptide and some others have been reported (reviewed in Ref. 40). These genes have promoter regions that can bind REST and thus would be expected to be downregulated. Yet, there are several considerations that need to be taken into account: (1) different RE1 sites have different REST affinities, thus the REST-dependent modulation of gene expression is concentration-dependent^27^; this may explain different efficacy (and perhaps kinetics) of suppression of potentially REST-sensitive genes during the development of a chronic pain state. (2) REST could suppress negative regulators of other genes; this, and the action of other transcription factors that might enhance these genes, may compensate for any repressive effect of REST. Finally, (3) there could be secondary mechanisms altering REST effect on some specific targets. In support of the last notion, increased phosphorylation of the methylated DNA binding protein MeCP2 in dorsal horn neurons in the chronic inflammatory pain model (CFA) has been reported.^16^ MeCP2 facilitates recruitment of CoREST complex by REST, but phosphorylation inhibits this activity and might disrupt REST-mediated repression of those genes, which are co-repressed by MeCP2.^40^ It seems therefore plausible that although primary function of REST is transcriptional repression, its upregulation in chronic pain may result in global multidirectional changes in expression of numerous genes. Consistent with this idea, recent study reported that inhibition of methyltransferase G9a (which is recruited to the repressor machinery orchestrated by REST) normalised the expression of over 600 genes whose expression was either downregulated or upregulated after nerve injury.^21^

Among the epigenetic changes observed within the peripheral afferents in chronic pain states, there is a general downregulation of expression of the K^+^ channel gene pool.^11,21,36^ This “negative drive” affects representative subunits of all major K^+^ channel families that are expressed in peripheral somatosensory system^11,36^ and includes tonically active K^+^ channels responsible for maintaining low resting excitability of peripheral afferents, specifically–M-type (Kv7, KCNQ) K^+^ channels^11,12,30^ and 2-pore (K2P) K^+^ channels.^23,37^ The case of M-type channels is perhaps the best evidenced, indeed these subthreshold, noninactivating K^+^ channels are increasingly recognised as being among the most important regulators of resting membrane potential and AP firing threshold in nociceptors (reviewed in Ref. 12). *Kcnq* genes encoding M channel subunits have functional RE1 binding sites, which are able to recruit REST, leading to the inhibition of transcription of *Kcnq2-5*.^18,25^ Moreover, overexpression of REST in DRG neurons robustly suppressed M current density and increased tonic excitability of these neurons.^25^ *Kcnq2*, *Kcnq3*, and *Kcnq5* are consistently downregulated in various chronic pain models (reviewed in Refs. 12,15), and downregulation of *Kcnq2* expression was correlated with the increased expression of REST after PSNL.^30^

Using ENCODE database, we identified a number of K^+^ channel genes that possess RE1 sites and were shown to bind REST within cells (suppl. Table 3, available at http://links.lww.com/PAIN/A830). Most intriguingly, a large number of these genes are expressed in nociceptive DRG neurons and virtually all of these RE1-containing K^+^ channel subunits were shown to be downregulated in DRG under induced chronic pain conditions. Thus, we hypothesize that (1) nerve injury–induced upregulation of REST is one of the major factors of neuropathic remodelling in DRG, and (2) downregulation of K^+^ channel expression by REST is a key mechanism underlying overexcitable phenotype of sensory neurons in chronic pain conditions. Consistent with this hypothesis, neuropathic injury-induced downregulation of 4 K^+^ channel genes *Kcna4*, *Kcnd4.2*, *Kcnq2*, and *Kcnma1* (coding for Kv1.4, Kv4.2, Kv7.2, and Slo1, respectively) was prevented by knockout of G9a,^21^ an enzyme recruited by REST upon its binding to RE1 sites of a REST target genes (reviewed in Ref. 27).

It has to be acknowledged that, at the final stages of preparation of this article, another study with partially overlapping findings has become available.^43^ Using different (noninducible) mouse line with sensory neuron-specific *Rest* knockout, Zhang and colleagues. have shown alleviation of neuropathic hyperalgesia in these animals; there was also a partial recovery of hyperalgesia after the siRNA downregulation of REST in DRG. These data independently support our findings. One difference between our data and the findings reported in Ref. 43 is that these authors found that knockout of *Rest* in their model only partially alleviated SNI-induced hyperalgesia; however, in our hands, deletion of *Rest* resulted in almost complete prevention of hyperalgesia in 3 different pain models (including SNI). Although reasons for this discrepancy are yet to be established, one hypothesis that could explain the difference is that Zhang and colleagues.^43^ used noninducible *Rest*-cKO; therefore, some developmental compensatory mechanisms could have partially offset the effect of *Rest* deletion in adult animals in that study. In the present investigation, an inducible and conditional *Rest* knockout has been used, and the deletion of *Rest* was performed immediately before the pain model establishment, minimizing the engagement of any long-term compensation mechanisms.

Importantly, most current approaches to tackle chronic pain aim to reduce the sensation of pain without the treatment of underlying pathology. Identification of REST as one of the key factors controlling neuropathic remodelling of DRG neurons may lead to identification of new therapeutic approaches whereby the very development of “painful” nerve phenotype can be prevented or reversed.

## Conflict of interest statement

The authors have no conflicts of interest to declare.

## Supplementary Material

SUPPLEMENTARY MATERIAL

## Appendix A. Supplemental digital content

Supplemental digital content associated with this article can be found online at http://links.lww.com/PAIN/A830, http://links.lww.com/PAIN/A826, http://links.lww.com/PAIN/A827, http://links.lww.com/PAIN/A828 and http://links.lww.com/PAIN/A829.
