# Supplementary material for: Repressor element 1–silencing transcription factor drives the development of chronic pain states
Source: Pain. 2019 Jun 14;160(10):2398–408. doi: 10.1097/j.pain.0000000000001633 (PMC6756259; doi:10.1097/j.pain.0000000000001633)
Supplement: SUPPLEMENTARY MATERIAL [file jop-160-2398-s001.docx]

**Supplemental Information**

**Supplemental Table 1.** List of primers used for single-cell RT-PCR

| **Gene/primer** | **Forward** | **Reverse** |
| --- | --- | --- |
| *Gapdh* inner | CCAGCCTCGTCCCGTAGACA | CGCTCCTGGAAGATGGTGAT |
| *Gapdh* outer | GAGAGGGAGGAGGGGAAATG | CTCGTGGTTCACACCCATCA |
| *Rest* inner | ACCACTACATGGCACACCTG | TTCTCACCTGAATGAGTCCGC |
| *Rest* outer | GAACCCCAGCCCGTATTTGA | TCTCACCTGAATGAGTCCGC |
| *Kcnq2* inner | AGGAAGCCGTTCTGTGTGAT | GCAGAGGAAGCCAATGTAC |
| *Kcnq2* outer | TCTCCTGCCTTGTGCTTTCT | GCATCTGCGTAGGTGTCAAA |
| *Kcnd3* inner | GAGGGGGTAGTGGGGAGTAA | CCCCTAATGCCAATCCCCT |
| *Kcnd3* outer | CACCAGTCGCTCCAGCCTTAAT | GGGCAGCTCTTGGTCTTGTG |
| *Oprm1* inner | ACTTCTGCATTGCCTTGGGT | AGAAAGCACATACCTGGTGGTT |
| *Oprm1* outer | TACAGGCAGGGGTCCATAGAT | TTCTCCAGTAACCGACCTCCT |
| *Scn10a* inner | ACCGACAATCAGAGCGAGGAG | ACAGACTAGAAATGGACAGAATCACC |
| *Scn10a* outer | TTGAAGAAGACACCGACGCA | TGTAAAACAGGCTTCGGGCT |

**Supplemental Table 2.** Incidence of detection of *Rest* and four of its target genes in DRG neurons as detected by single-cell RT-PCR in tamoxifen-injected *Rest*^loxP/loxP^/WT mice in control conditions, four weeks after the SNI injury and in the tamoxifen-injected *Rest*^loxP/loxP^/AvCreER-T2 mice after the SNI injury.

| **Gene name** | | ***Rest*^loxP/loxP^/WT**  **(control)** | ***Rest*^loxP/loxP^/WT+SNI** | ***Rest*^loxP/loxP^/AvCreER-T2+SNI** |
| --- | --- | --- | --- | --- |
| Total number of neurons analysed | | 79 | 89 | 79 |
| Number of positive cells (%) | *Gapdh* | 79 (100) | 57 (64)*** | 79 (100)^###^ |
|  | *Rest* | 15 (19) | 48 (54)*** | 4 (5)^###^ |
|  | *Kcnq2* | 46 (58) | 19 (21)*** | 30 (38)^#^ |
|  | *Kcnd3* | 48 (61) | 29 (33)*** | 52 (66)^###^ |
|  | *Oprm1* | 32 (41) | 24 (27) | 25 (32) |
|  | *Scn10a* | 18 (23) | 14 (16) | 18 (23) |

*** Significantly different from control; Fisher’s exact test, p<0.001

^#^,^###^ Significantly different from tamoxifen-injected *Rest*^loxP/loxP^/WT mice after the SNI injury; Fisher’s exact test, p<0.05 or p<0.001

**Supplemental Table 3.** RE1-containing K^+^ channel genes, their expression in sensory afferents and chronic pain associated downregulation.

| **Gene name**^1^ | **Subunit** | **Expression in nociceptors**^2^ | **Downregulated in chronic pain model(s)**^2^ |
| --- | --- | --- | --- |
| KCNA2 | Kv1.2 | **++** | Yes |
| KCNA4 | Kv1.4 | **+++** | Yes |
| KCNC1 | Kv3.1 | **+** | unknown |
| KCNC3 | Kv3.3 | **-** | unknown |
| KCNC4 | Kv3.4 | **+++** | Yes |
| KCND3 | Kv4.3 | **+++** | Yes |
| KCND2 | Kv4.2 | **+** | Yes |
| KCNH1 | EAG | **-** | unknown |
| KCNH2 | HERG | **-** | unknown |
| KCNH4 | ELK1 | **-** | unknown |
| KCNK9 | TASK3 | **+** | Yes |
| KCNMA1 | KCa1.1 (SLO1) | **++** | Yes |
| KCNN4 | KCa3.1 (SK4) | **+**^3^ | unknown |
| KCNQ2 | Kv7.2 | **+++** | Yes |
| KCNQ3 | Kv7.3 | **+++** | Yes |
| KCNQ5 | Kv7.5 | **+++** | Yes |
| KCNAB2 | BETA-2 | **++** | unknown |
| KCNIP2 | KCHIP2 | **+++**^4^ | unknown |
| KCNIP4 | KCHIP4 | **-** | unknown |

^1^RE1-containing K^+^ channel genes for which REST binding has been identified by Transcription Factor ChIP-seq Uniform Peaks from ENCODE/Analysis (<http://genome-euro.ucsc.edu>)

^2^Unless indicated otherwise, data are from Du X & Gamper N (2013) Potassium channels in peripheral pain pathways: expression, function and therapeutic potential. Curr Neuropharmacol 11:621-640.

^3^Lu R, et al. (2017) KCa3.1 channels modulate the processing of noxious chemical stimuli in mice. Neuropharmacology 125:386-395.

^4^Kuo YL, et al. (2017) K^+^ Channel Modulatory Subunits KChIP and DPP Participate in Kv4-Mediated Mechanical Pain Control. J Neurosci 37:4391-4404.


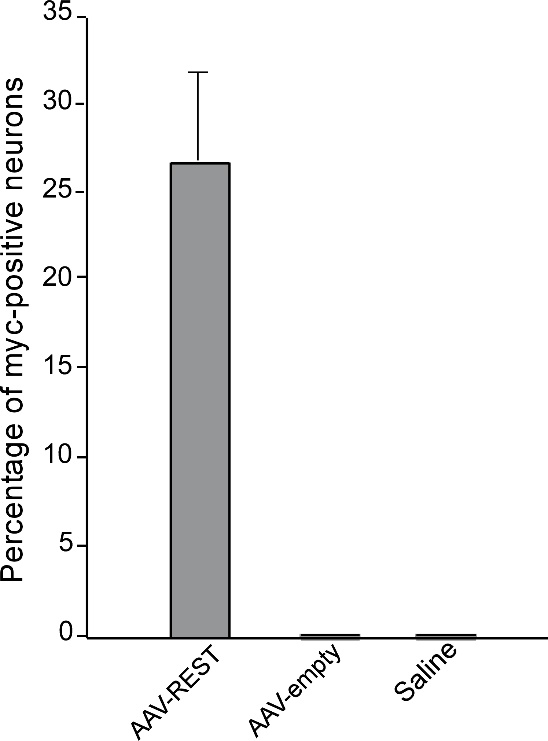


**Supplemental Figure 1.** Quantification of immunohistochemical analysis of Myc expression in L4 DRG of mice DRG-injected with AAV2/9-REST, empty AAV2/9 particles or saline (example images are presented in Fig. 1C); for each conditions 3 mice, 5-6 sections were analysed.


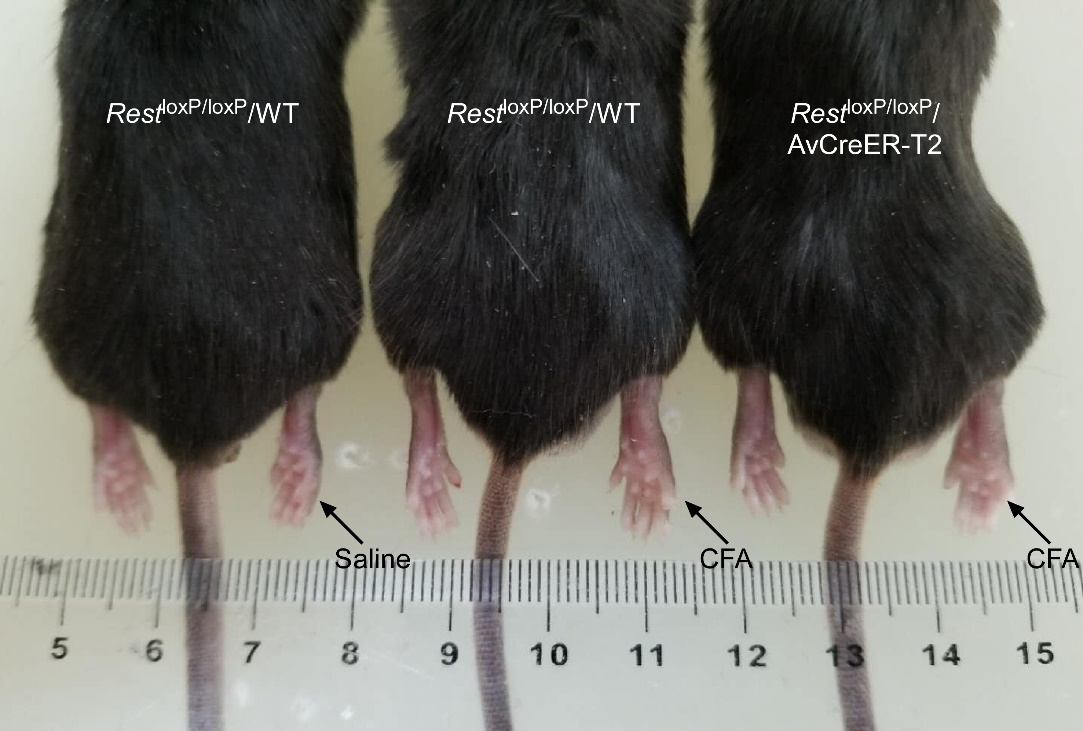


**Supplemental Figure 2. Deletion of Rest does not affect paw edema. H**ind-paw injection of the Complete Freund’s Adjuvant (CFA, 20 μl) produced similar degree of paw swelling in the tamoxifen-injected *Rest*^loxP/loxP^/AvCreER-T2 and *Rest*^loxP/loxP^/WT mice; injection of saline produced no swelling. Photographs are taken 2 weeks after the CFA injection.


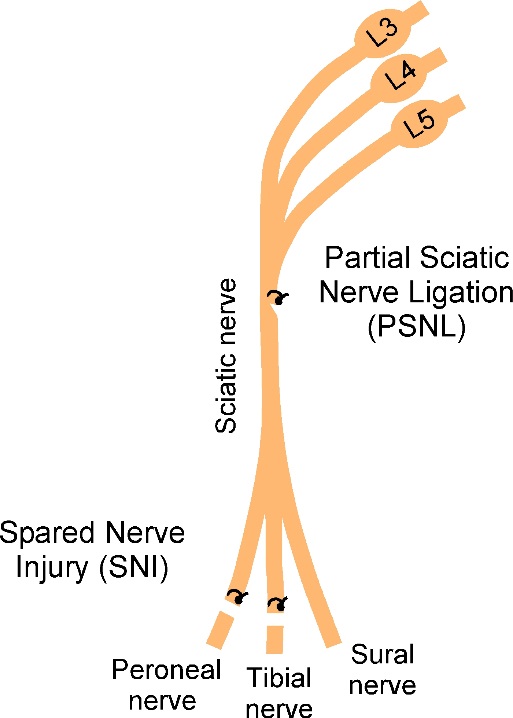


**Supplemental Figure 3.** Schematic of neuropathic pain models used.


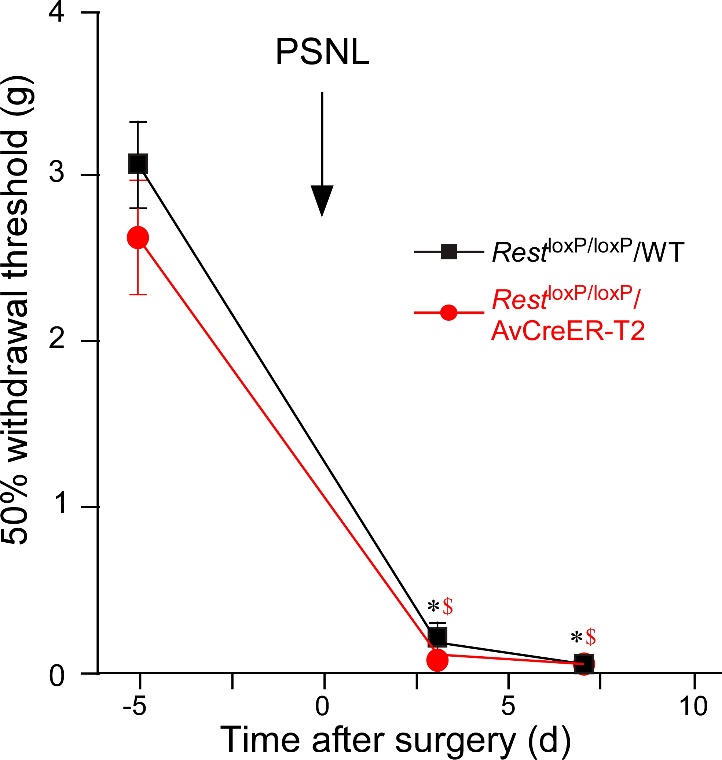


**Supplemental Figure 4.** In the absence of tamoxifen *Rest*^loxP/loxP^/AvCreER-T2 mice have normal mechanical threshold and develop hyperalgesia following partial sciatic nerve ligation (PSNL) similarly to the WT littermates. *Rest*^loxP/loxP^/AvCreER-T2 (red symbols, lines; n=10); *Rest*^loxP/loxP^/WT (black symbols, lines; n=10).^*^,^$^ different from the pre-injury measurements in the same animal; P < 0.05 (two-way repeated measures ANOVA with Tukey post-hoc test).

**Supplemental Movie 1.** Wild-type mice *in vivo* injected with AAV2/9 REST into the right L4 DRG display paw dragging and change of gait. Video was recorded on day 34 after the injection.

**Supplemental Movie 2.** Control wild-type mice *in vivo* injected with empty AAV2/9 into the right L4 DRG display normal gait. Video was recorded on day 34 after the injection.

**Supplemental Movie 3.** Tamoxifen-injected *Rest*^loxP/loxP^/WT (control) mice after SNI displayed dragging of injured (right) paw and changed gait. Video was recorded on day 21 after the surgery.

**Supplemental Movie 4.** Tamoxifen-injected *Rest*^loxP/loxP^/AvCreER-T2 mice after SNI displayed nearly normal gait and no dragging of the injured (right) paw. Video was recorded on day 21 after the surgery.
